# Supplementary figures and images for: A Novel 10-Base Pair Deletion in the First Exon of GmHY2a Promotes Hypocotyl Elongation, Induces Early Maturation, and Impairs Photosynthetic Performance in Soybean
Source: Int J Mol Sci. 2024 Jun 12;25(12):6483. doi: 10.3390/ijms25126483 (PMC11203641; doi:10.3390/ijms25126483)

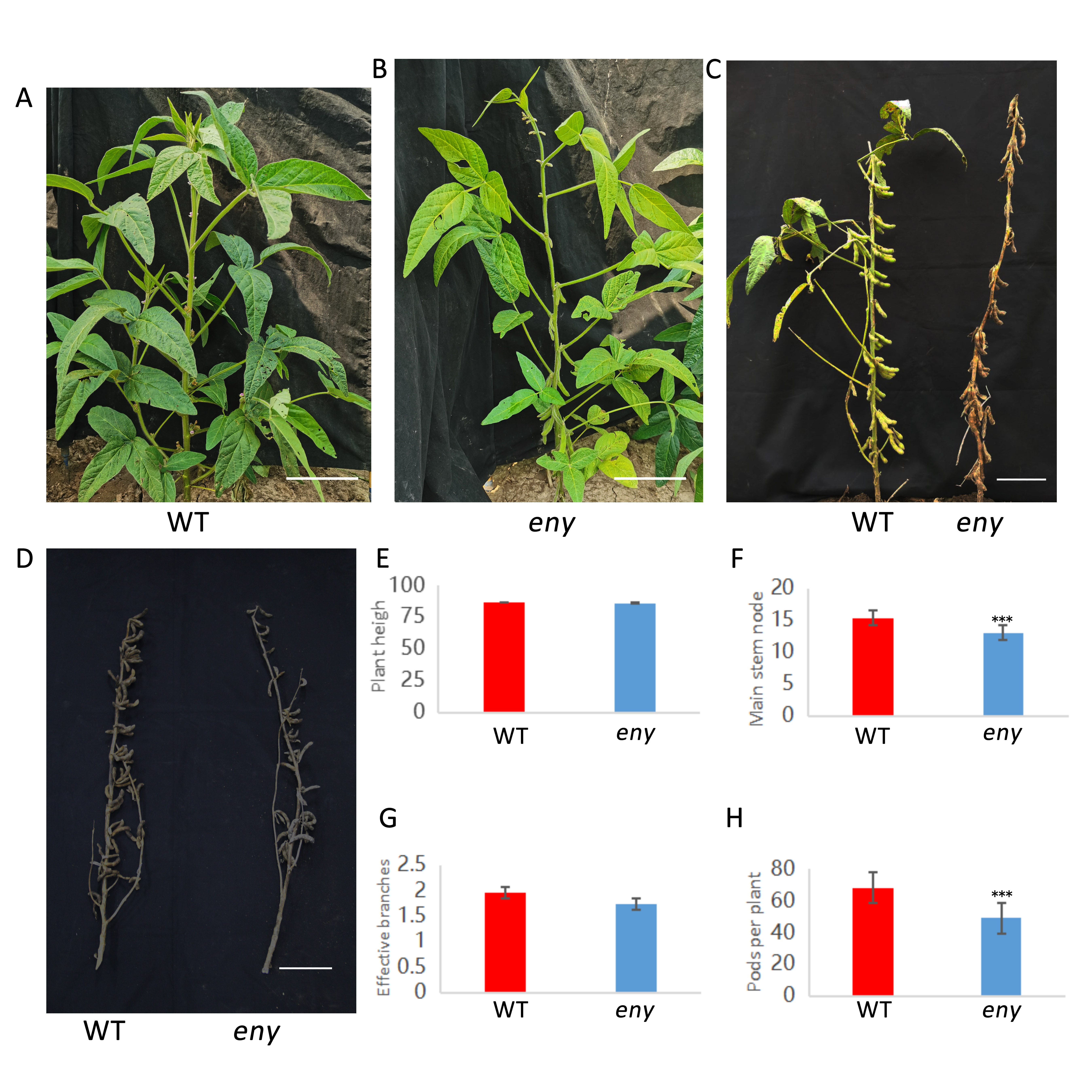

Supplement: Supplementary file 1 [file ijms-25-06483-s001.zip › Supplemental Figure S1.jpg]

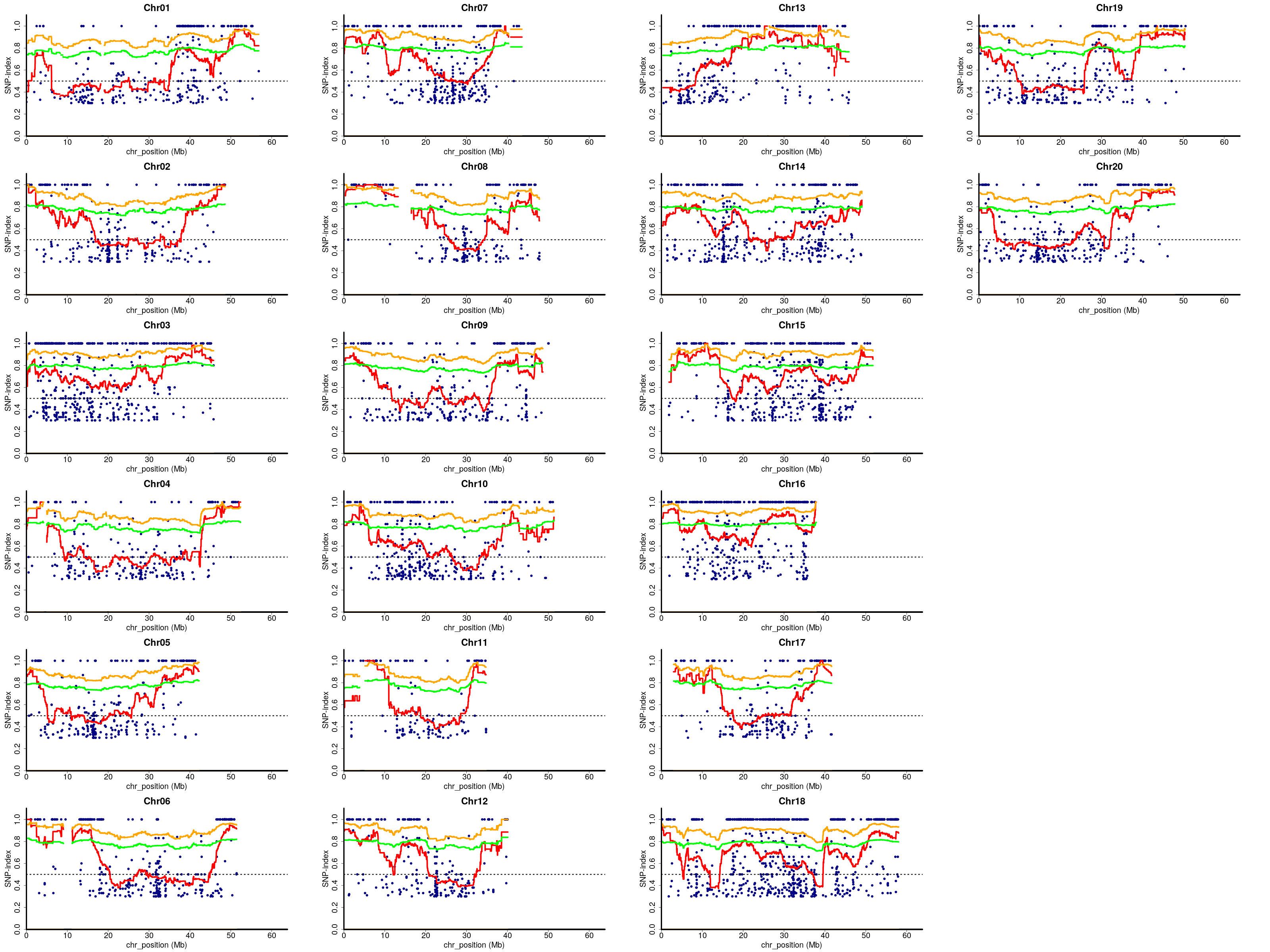

Supplement: Supplementary file 1 [file ijms-25-06483-s001.zip › Supplemental Figure S2 mask5_mybulk_q30p90_filtered_pvalue_sldwnd4M10K_cov4_co5.png]

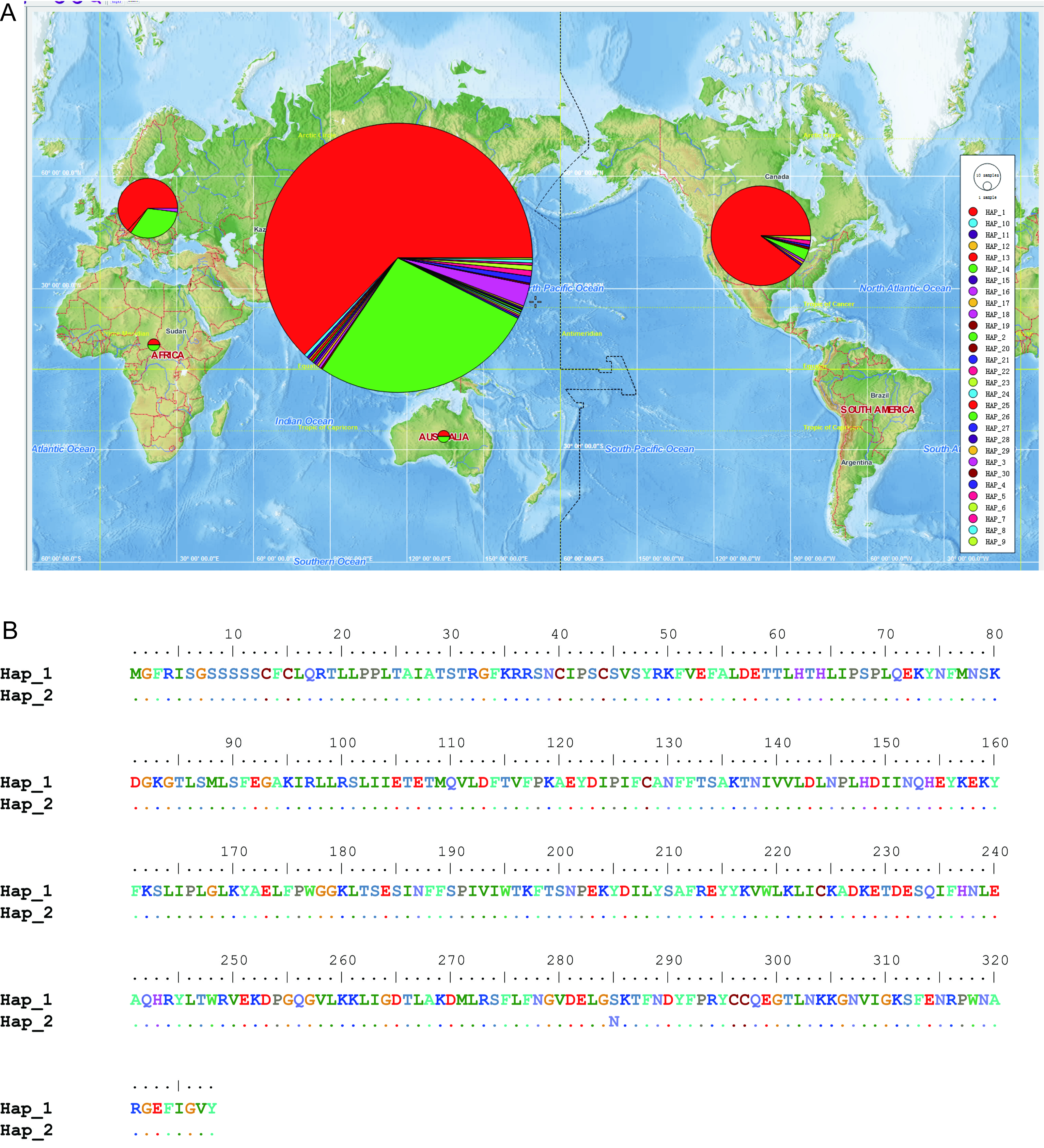

Supplement: Supplementary file 1 [file ijms-25-06483-s001.zip › Supplemental Figure S3 haplotype.tif]

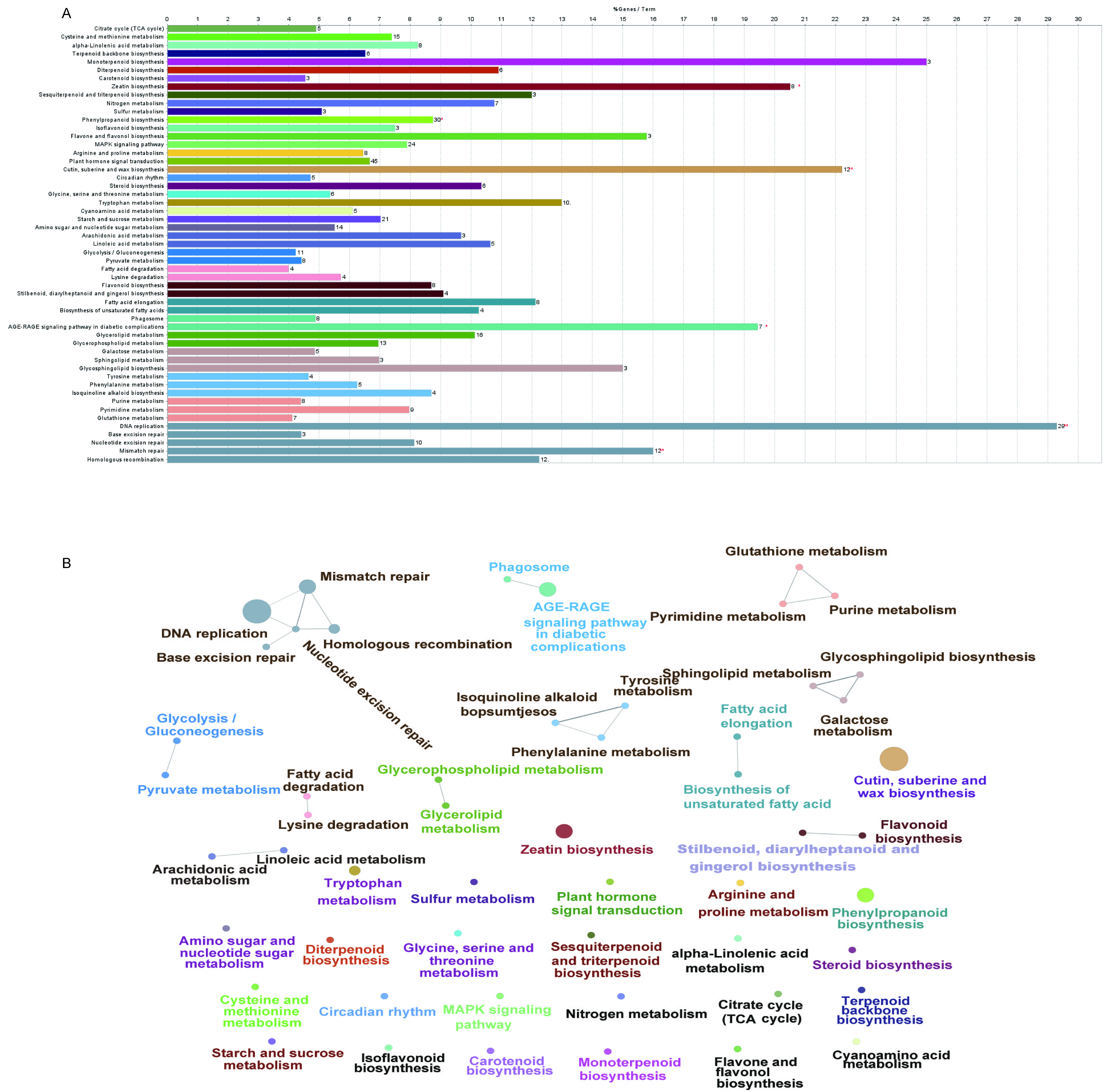

Supplement: Supplementary file 1 [file ijms-25-06483-s001.zip › Supplemental Figure S4 KEGG original.jpg]
